# Supplementary material for: A DSM-5 AMPD and ICD-11 compatible measure for an early identification of personality disorders in adolescence–LoPF-Q 12–18 latent structure and short form
Source: PLoS One. 2022 Sep 21;17(9):e0269327. doi: 10.1371/journal.pone.0269327 (PMC9491532; doi:10.1371/journal.pone.0269327)
Supplement: S1 Table — (PDF) [file pone.0269327.s001.pdf]

LoPF-Q 12-18 model to structure the four domains of personality functioning (PFs) into scales, subscales, and facets. To clarify the integrated aspects of impairment, the pathological pole of the facets and marker items are given. (-) = Item formulation represents non-pathological functioning.

**PF1: Identity:** Self-integration vs. Diffusion<br>Ego-Strength vs. Ego-Weakness - “I don’t know who I am”
**1.1. Continuity**<br>(facets (pathological pole): lack of consolidating perspectives, roles, and emotional self-experience)<br>*“I can imagine the kind of person that I will be in a few years.” (-); “I have nothing in common with the most people my age.”; “Sometimes I have strong feelings without knowing where they come from.”*
**1.2 Coherence**<br>(facets (pathological pole): inconsistent self-images, lack of autonomy, diffuse representations)<br>*“I often feel lost, as if I had no clear inner self.”; “When I’m alone I feel helpless.”; “I am confused about what kind of person I really am.”*
**PF2: Self-direction**: Self-realization vs. Conative pathology<br>Personally effective vs. ineffective - “I don’t know what I want”
**2.1. Self-congruence**<br>(facets (pathological pole): impaired self-acceptance, self-reflection, self-regulation)<br>*“I often have a bad opinion about myself.”; “I am often unable to explain to myself why I did something.”; “When I am upset, my emotions escalate until I flip out or break down.”*
**2.2 Purposefulness**<br>(facets (pathological pole): resignation, lack of direction, passivity, self-sabotage)<br>*“Often I don´t know what to do with my life.”; “Often I simply don’t have energy to push my agenda.”; “I have difficulties to reach the goals that I set for myself.”*
**PF3: Empathy**: Prosocial vs. impaired societal functioning<br>Prosocial vs. unsocial - “The other is just a tool”
**3.1. Perspective taking**<br>(facets (pathological pole): impaired emotionality, cognitive empathy / considering social causality)<br>*“Others perceive me as unfeeling.”; “I often don’t understand other people’s reactions to my behavior.”; ”Often others experience my behavior quite different from how it was meant.”*
**3.2 Prosociality**<br>(facets (pathological pole): uncooperative / bitter, reckless / callous-manipulative)<br>*“It gives me a good feeling to point out others’ mistakes.”; ”If I need something, I don’t care if I harm somebody else to get it.”; “If someone allows me to treat them badly, they don’t deserve any better.”*
**PF4: Intimacy**: Personal vs. impaired personal relationships<br>Trustful vs. withdrawn - “The other is dangerous”
**4.1. Capacity for close relationships**<br>(facets (pathological pole): impaired tolerance of closeness, emotional openness, trust)<br>*“I prefer others to not get too close to me.”; “I get uncomfortable when others notice how I am feeling.”; “I am often worried about getting hurt in friendships.”*
**4.2 Reciprocity**<br>(facets (pathological pole): superficial, detached, solitary, lack of mutuality)<br>*“I feel like I don’t really belong with anyone.”; “I feel uncomfortable when a friendship becomes too close.”; “It is important for me to get to know my friends very well, so that we can be "real friends".” (-)*

© 2018 academic-tests. All rights reserved.

Reference: Sharp C & Vanwoerden S (2018). *Culture-adapted version English USA of the self-report questionnaire LoPF-Q 12-18 (Levels of Personality Functioning Questionnaire; authors Goth & Schmeck) – Short manual.* Offenbach: academic-tests. https://academic-tests.com
