# Supplementary material for: A DSM-5 AMPD and ICD-11 compatible measure for an early identification of personality disorders in adolescence–LoPF-Q 12–18 latent structure and short form
Source: PLoS One. 2022 Sep 21;17(9):e0269327. doi: 10.1371/journal.pone.0269327 (PMC9491532; doi:10.1371/journal.pone.0269327)
Supplement: S2 Table — (PDF) [file pone.0269327.s002.pdf]

Supplemental table. Energy consumption and CPU time for item permutation - Need for an optimised item selection algorithm

	Different item combinations	Estimated CPU time [1]	Energy required [2]	CO2 Emission [3]
Random selection of 20 / 97 items [a]	2.72×10^{20}	~ twenty trillion years	2.8 million TWh	26 thousand Tt
Random selection of 4 items for each domain [b]	6.25×10^{18}	~500 billion years	65,000 TWh	605 Tt
100,000 random combinations of items (5 per domain)	100,000	2.89 days	~1 kWh	9.7g
Ant colony optimization		~ 4 hours	60Wh	0.6g

Note. Possible combinations were calculated using formulae [a] $\binom{97}{20}$ and [b] $\binom{26}{5} \times \binom{23}{5} \times \binom{25}{5} \times \binom{25}{5}$

[1] CPU time was assessed using the R function proc.time. For a standard home computer (Core(TM) i7-8550U CPU 1.99 GHz) the average CPU time per item combination was 2.5 sec.

[2] Intel referenced power consumption of the aforementioned processor as 15W

[3] For Switzerland $C0_2$ emissions have been estimated as 9.3g per produced kWh.
