# Supplementary material for: A DSM-5 AMPD and ICD-11 compatible measure for an early identification of personality disorders in adolescence–LoPF-Q 12–18 latent structure and short form
Source: PLoS One. 2022 Sep 21;17(9):e0269327. doi: 10.1371/journal.pone.0269327 (PMC9491532; doi:10.1371/journal.pone.0269327)
Supplement: S1 File — (PDF) [file pone.0269327.s004.pdf]

Formulas for calculating pheromones

The final pheromone was calculated as:

$$Pheromone = \varphi_{Fit} + \varphi_{Rel} + \varphi_{Val}$$

Where φ_{Fit} was calculated based on both fit indices:

$$\varphi_{Fit} = \frac{\varphi_{CFI} += \varphi_{RMSEA}}{2}$$

Where φ_{CFI} was modelled to increase with higher values, whereas φ_{RMSEA} was modelled to decrease with higher values:

$$\varphi_{CFI} = \frac{1}{1+e^{(0.95-CFI)\cdot 100}}$$

$$\varphi_{RMSEA} = 1 - \frac{1}{1+e^{(0.05-RMSEA)\cdot 100}}$$

Where φ_{Rel} was calculated based on both reliability indices, i.e. McDonald's Omega and minimal factor loadings:

$$\varphi_{Rel} = \frac{\varphi_{OMEGA} += \varphi_{LAMBDA}}{2}$$

Where φ_{OMEGA} was modelled to increase logistically with higher values, and φ_{LAMBDA} was based on the minimal factor loading (λ) of the selected n items:

$$\varphi_{OMEGA} = \frac{1}{1+e^{(0.7-OMEGA)\cdot 100}}$$

$$\varphi_{LAMBDA} = \frac{1}{1+e^{(0.5-\lambda)\cdot 100}}$$

Where φ_{Val} was calculated based on the external validity criterion, i.e. the logistic transformation of the adjusted R^2 derived from a regression model in which the external criterion was the dependent variable.

$$\varphi_{R^2} = \frac{1}{1 + e^{(0.4-R^2)\cdot 100}}$$

The figure below illustrates logistic functions that were used to transform measures of model fit (CFI, RMSEA), reliability (Mc Donald's Omega), external validity (adjusted r squared of a linear regression model predicting personality disorder vs. no personality disorder), as well as factor saturation (minimal factor loadings).

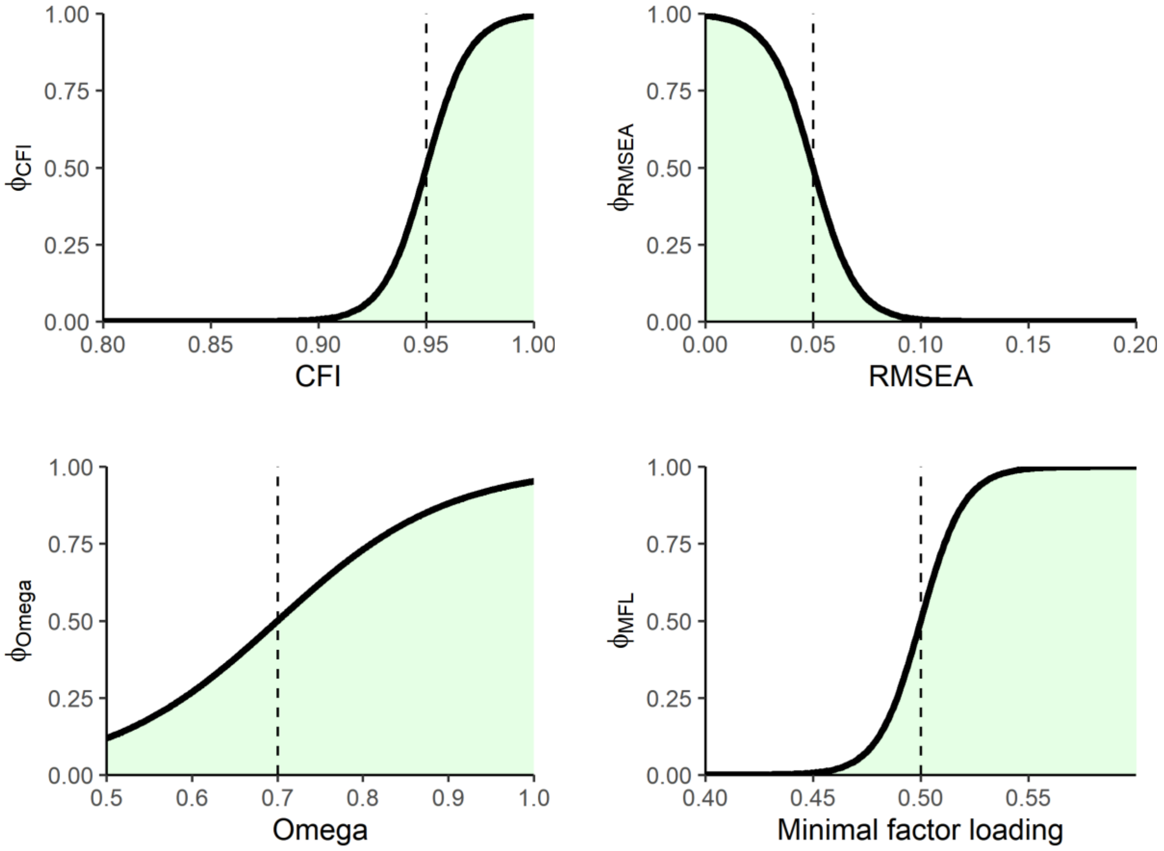

1.00
0.75
0.50
0.25
0.00
φCFI
0.80
0.85
0.90
0.95
1.00
CFI
φRMSEA
0.00
0.05
0.10
0.15
0.20
RMSEA
φOmega
0.5
0.6
0.7
0.8
0.9
1.0
Omega
φMFL
0.40
0.45
0.50
0.55
Minimal factor loading
